# THE MAYAK WORKER DOSIMETRY SYSTEM (MWDS-2013): AN INTRODUCTION TO THE DOCUMENTATION

**DOI:** 10.1093/rpd/ncx020

**Published:** 2017-03-17

**Authors:** B A Napier

**Affiliations:** 1 Energy and Environment Directorate, Pacific Northwest National Laboratory, Richland, WA, USA

## Abstract

The reconstruction of radiation doses to Mayak Production Association workers in central Russia supports radiation epidemiological studies for the U.S.-Russian Joint Coordinating Committee on Radiation Effects Research. The most recent version of the dosimetry was performed with the Mayak Worker Dosimetry System-2013. This introduction outlines the logic and general content of the series of articles presented in this issue of *Radiation Protection Dosimetry*. The articles summarize the models, describe the basis for most of the key decisions made in developing the models and present an overview of the results.

## INTRODUCTION

The Mayak Worker Dosimetry project, which is Project 2.4 of the Joint Coordinating Committee on Radiation Effects Research^(1)^ (JCCRER), is providing estimates of radiation dose from occupational exposure to direct radiation and inhaled materials at the Mayak Production Association (PA). The Mayak Worker Dosimetry System has been developed over many years’ collaboration between Russian and American scientists with input from many other countries and organizations. The most recent version of the system is MWDS-2013. To promote transparency and understanding for the radiation protection community, it was decided to document the system as completely as possible in the open literature. The series of articles in this issue of *Radiation Protection Dosimetry* describes the system and the logic of the choices made in its development. Many model parameters were developed from original research within the project; these are also described.

## THE ARTICLES IN THIS SERIES

The MWDS-2013 system consists of two major components. The first is the set of models for estimating dose from inhaled materials, which collectively is named PANDORA. The second is the set of models for estimating dose from external sources of irradiation (which currently does not have a separate name; it is derived fairly directly from earlier versions of the MWDS system^(2)^).

The PANDORA system incorporates a Bayesian approach to determining plutonium intake based upon historical urinalysis samples. This model is described in a detailed fashion by Birchall *et al*. in the article *The Mayak Worker Dosimetry System (MWDS-2013) for Internally Deposited Plutonium: An Overview*^(3)^. Because of the necessarily complex nature of this description, much of the supporting detail is provided in the remaining papers of the series.

The supporting detail covers several different general topics. Because the model is focused on the inhalation pathway, several key model parameter choices relate to the lung model. A proper description of the behavior of inhaled plutonium in the lung is critical to the MWDS-2013 modeling effort. The project has spent a substantial amount of time and effort to investigate the behavior of plutonium particles in human lungs. Two key concepts are the possibility for binding of material to lung tissue and the rate at which particles dissolve.

The lung model includes consideration of a possible bound fraction, which represents radionuclides that have become chemically bound in the lungs following dissolution of particulates in lung fluid. Bound radionuclides are not subject to particle transport clearance but can be absorbed to blood. The occurrence of long-term binding of plutonium can greatly increase lung doses, particularly if it occurs in the bronchial and bronchiolar regions. The issue of binding is addressed in four articles. Puncher *et al*.^(4)^ review the historic experiments in which beagle dogs were exposed to plutonium nitrate aerosols. A Bayesian analysis is used to look for evidence of long-term retention in lungs. The three articles using human evidence are led by Tolmachev *et al*.^(5)^ with a description of a study of long-term retention of plutonium nitrate in the lungs of a US Transuranium and Uranium Registries donor. For the first time, plutonium distribution in human upper airways was investigated based on the dosimetric structure of the human respiratory tract proposed by the International Commission on Radiological Protection (ICRP)^(6)^. The data collected in the Tolmachev *et al*.^(5)^ study is further analyzed by Puncher *et al*.^(7)^ using a novel Bayesian analysis to determine the value of the bound fraction parameter. This technique is then extended to the Mayak cohort by Puncher *et al*.^(8)^ to determine the bound fraction applicable to MWDS-2013 specifically for the workers being studied.

Lung doses resulting from inhalation of plutonium aerosols are highly dependent on the assumed rate of particle clearance, which occurs by two competing processes—physical transport clearance to the alimentary tract and to the thoracic lymph nodes; and clearance to systemic tissues by dissolution of particles in lung fluid followed by uptake to blood (a process collectively known as absorption). Puncher *et al*.^(9)^ use a Bayesian approach to estimate the slow rate of dissolution of both oxides and nitrates in the lungs of Mayak PA workers.

The inhalation dose estimates of MWDS-2013 are based on actual bioassay measurements (urinalysis and autopsy sampling) for the workers. In order to estimate doses for workers exposed to plutonium, it is also necessary to make assumptions about the time course of intake. Sokolova *et al*.^(10)^ examine records from workplace air sampling, personnel biophysical examinations and autopsy data from former Mayak PA workers to develop a three-step function that describes the general conditions of exposure over time at Mayak. This function is convoluted with each individual worker's work history to determine the actual exposure ‘scenario’ for that worker.

Once inhaled, plutonium may be transported to organs other than the lungs through the bloodstream. MWDS-2013 uses a model developed by Leggett *et al*.^(11)^ to represent the biokinetic behavior of plutonium after uptake to blood. Birchall *et al*.^(12)^ use measurement data from Mayak PA workers to validate the use of this biokinetic model.

The biokinetic models provide time-integrals of the activity in workers’ organs and tissues; this is converted to radiation dose. Previous epidemiological studies involving Mayak PA workers have used a lung dose calculated as the total energy deposited in the lungs divided by the mass. In the MWDS-2013, lung dose is calculated as an average of the three absorbed doses to the bronchial, the bronchiolar and the alveolar regions, for reasons describe by Birchall and Marsh^(13)^. Similarly, for other organs, the MWDS-2013 doses are calculated by dividing the energy by a population average organ mass. The reasons for departing from previous methodologies are described by Birchall and Sokolova^(14)^.

The dose estimates of MWDS-2013 are firmly based on bioassay measurements made of workers’ urine and of autopsy samples. The Bayesian approach implemented in the PANDORA system incorporates the uncertainties of the individual measurements along with the more global uncertainties of the models and their parameters.

Urine analyses were made at different times over the Mayak PA history using different techniques and accounting for different factors (e.g. whether or not creatinine is measured, the volume of the sample, whether DTPA was administered, etc.) and these variables affect not only the uncertainty in the estimated doses but also the central values of the doses themselves. Vostrotin *et al*.^(15)^ describe and justify how the parameters of the uncertainty distribution are derived from the raw data of each urine measurement technique.

Other bioassay data come from autopsy samples of bone and lung. Two to nine bone samples per subject were obtained at autopsies conducted from the mid-1950s until 2013; plutonium was measured using alpha-radiometry up to 2000 and later by alpha-spectroscopy. Suslova *et al*.^(16)^ describe how full skeletal burdens of plutonium were estimated from a limited number of samples and how the approach was verified using data from whole-body donors from the US Transuranium and Uranium Registries. In a parallel fashion, historical autopsies also yielded samples from one to all five lung lobes and one to all three important thoracic lymph node types. Sokolova *et al*.^(17)^ describe a method to estimate organ plutonium burden for cases where incomplete lung or lymph node samples were obtained—one to four lung lobe samples and one to two thoracic lymph nodes. The method was validated using individual measurement data from 259 Mayak PA autopsy cases with complete lung and lymph samples.

MWDS-2013 deals directly with uncertainty in the model parameters and other inputs, and differentiates parameters that are considered to be shared and unshared. Birchall *et al*.^(18)^ describe the way in which uncertainty is dealt with to produce the final output represented by multiple hyper-realization of organ doses including:

- Application of the WeLMoS^(19, 20)^ method to calculate Bayesian posterior probability distributions of organ doses.
- Extension of the WeLMoS method for dealing with multiple intake regimes.
- How shared (unknown, but having the same value for all workers) and unshared (unknown, but having different values between workers) parameters are dealt with using a multiple realization method.
- A practical algorithm for the generation of multiple hyper-realizations.
- How to deal with uncertainty in the intake and the intake regime.

The output hyper-realizations of PANDORA incorporate the overall uncertainty of the doses, but result in essentially one million results per individual. In order to make preliminary epidemiological analyses tractable, and also for consistency with the external doses, it was required to convert the hyper-realizations to realizations using a method described by Birchall and Puncher^(21)^.

The calculation of internal doses with PANDORA for MWDS-2013 involved extensive computational resources due to the complexity and sheer number of calculations required. Zhdanov *et al*.^(22)^ describe the hardware components and computational approaches required to make the calculation manageable. A single run of the code for the entire cohort took about 47 days.

It was considered imperative that the results be extensively tested for quality. Two articles describe the two phases of the quality assurance (QA) process. The first phase compared the outputs of PANDORA to the similar but independently developed code IMBA Professional Plus^(23)^. Dorrian *et al*.^(24)^ report that calculated values of absorbed doses, intakes, organ burdens and urinary excretion agreed to within 1% for fixed sets of input parameters, with differences attributed to minor approximations made with the IMBA code. Vostrotin *et al*.^(25)^ describe the second phase of QA in which the full distributions were separately calculated on plutonium inhalation intake; and on lifetime measurements of plutonium activity in daily urine and postmortem measurements in lungs, thoracic lymph nodes, liver and skeleton. Estimates of geometric mean and geometric standard deviation of annual regionally weighted lung dose and bone surface dose were compared.

The final calculations for all years and all organs for nearly 8000 Mayak PA workers generated output files approaching terabyte magnitudes. The results of the internal dose calculations are summarized by Vostrotin *et al*.^(26)^ The results are characterized and compared with those of previous Mayak PA dosimetry systems.

The external dose estimation process for MWDS-2013 was similar but not as extensive. The staff members of the Mayak PA itself were not able to work with the JCCRER team for the majority of the research period. As a result, the methods of earlier external dose modeling were adapted and expanded to account for uncertainties in the exposure scenarios, measurements and other parameters. Napier *et al*.^(27)^ describe the calculation methods and parameter distributions incorporated in the external irradiation components of MWDS-2013. The article also summarizes the results of external dose estimation from occupational and medical exposures for more than 25 000 MPA workers.

## CONCLUSIONS

A multi-year dosimetry effort culminates in this issue of *Radiation Protection Dosimetry*. The Mayak PA worker MWDS-2013 internal and external dose estimates and their associated uncertainties have been transferred to project biostatisticians for epidemiological analyses.

Compared with previous studies, there are many differences between the model structures and methodology employed in this study. These are described in the individual articles. However, the main difference is that MWDS-2013 takes explicit account of uncertainties in model parameters. Previous studies used models with discrete model parameters. Probability distributions are specified for important model parameters which reflect the uncertainty in the parameter value. The outputs of the system are thus distributions of dose rather than single values, with appropriate consideration of shared and unshared parameters for incorporation in advanced epidemiological modeling.
